# Is It Possible to Thrive During a Pandemic?

**DOI:** 10.3389/fpsyg.2022.759665

**Published:** 2022-01-31

**Authors:** Geoffrey V. Henderson, Andrew J. Elliot

**Affiliations:** ^1^Department of Physical Medicine and Rehabilitation, Syracuse DVAMC and SUNY Upstate Medical Center, Syracuse, NY, United States; ^2^Department of Psychology, University of Rochester, Rochester, NY, United States

**Keywords:** approach-avoidance motivation, goals, sub-goals, hierarchical model, COVID-19

## Introduction

SARS-CoV-2—the virus that causes COVID-19—emerged in the waning months of 2019 and spread globally thereafter (Timeline of WHO’s Response to COVID-19, 2020). The virus and its variants continue to infect record numbers, killing over five million people worldwide (WHO Coronavirus (COVID-19) Dashboard, 2021). Individuals and societies have sought behavioral interventions to minimize exposure and spread of the illness, as effective vaccines have only recently become available, and vaccine distribution has been variable.

Motivation science can offer guidance about goal-directed behavior during a pandemic. Goals, which are consciously selected, play a key role in regulating and guiding behavior (Elliot and Fryer, 2008). A foundational precept of behavior involves moving toward a positive object (approach) or moving away from a negative object (avoidance) (Elliot and Covington, 2001). Here, object is used broadly to refer to an object, event, or circumstance. This approach-avoidance dichotomy is a fundamental distinction in motivation, and proves a useful framework to examine the costs and benefits of behavioral responses to the pandemic.

## Avoiding COVID-19

Given that the pandemic is a markedly negative event, caused by a deadly contagion, avoidance is prudent—avoiding this negative “object” is the preferred choice (Henderson and Elliot, 2021). Avoidance motivation may be seen as the appropriate choice when survival is at stake (which is arguably the case during a pandemic). Beyond supporting survival, avoidance motivation can offer other benefits: it evokes persistent, vigilant information processing that can facilitate performance on certain types of tasks. Also, it prompts the selective recruitment and expenditure of cognitive resources in certain situations that can facilitate self-regulation (Roskes et al., 2013). Nonetheless, the activation and enactment of avoidance motivation comes at a steep cost: it saps energy, depletes well-being, and undermines goal attainment, especially in the long-run (Oertig et al., 2013). We will term these costs an “avoidance tax,” as these symptoms are psychologically taxing and a consistent feature of avoidance motivation. The question then becomes, how can one best pursue avoiding COVID-19 (or a subsequent deadly disease), given that avoidance is the primary (and taxing) strategy?

To explore this possibility, it is important to highlight that goals exist in a hierarchy, with superordinate (primary) goals supported by subordinate (secondary) goals (Elliot, 2006). Goal seekers are often encouraged to link large superordinate goals to smaller, more manageable “chunks” or subordinate goals (hereafter referred to as sub-goals). Examining these sub-goals may yield insight into the type and nature of sub-goals that best support superordinate goal attainment. Moreover, the hierarchical structure allows the possibility of using approach sub-goals to achieve the primary avoidance aim (approach IN ORDER TO avoid) (Elliot and Thrash, 2001). Approach motivation typically boosts energy, increases well-being, and facilitates goal attainment. That is, approach motivation helps individuals thrive (Elliot and Gable, 2019), and approach sub-goals can potentially be co-opted to promote avoidance superordinate goals and mitigate avoidance costs.

## Assist and Address Sub-goals

To understand goal attainment during the current difficult and demanding circumstances, it is important to distinguish between types of sub-goals and to delineate how each type can be utilized to promote goal attainment and well-being. We propose that two types of sub-goals can be identified: assist and address sub-goals.

*Assist* sub-goals are those that directly assist in attaining the superordinate goal. Assist sub-goals are tightly aligned with the superordinate goal; the overall aim is the same, so to speak, between superordinate and sub-goals. These sub-goals focus on components of the whole or promote goal attainment by varied mechanisms or tactics. Assist sub-goals are the type of sub-goal that has been widely conceptualized and studied in the literature on goal hierarchies (for reviews, see Ford, 1992; Austin and Vancouver, 1996; Carver and Scheier, 1998; Gozli and Dolcini, 2018).

*Address* sub-goals are those that address the limitations and implications of superordinate goal pursuit. For example, the pitfalls of avoidance goal pursuit include waning energy, depleted well-being, and flagging goal commitment. Sub-goals that address these downsides can boost goal attainment by reducing these limitations; these sub-goals do not directly facilitate the superordinate goal (as do assist sub-goals), but instead address the negative implications of superordinate goal pursuit. Address sub-goals are a novel type of sub-goal that we are proposing herein; empirical work has yet to be conducted on this construct.

Assist and address sub-goals can be approach- or avoidance-oriented; sub-goals do not need to match the valence of the superordinate goal. See Table 1 for a summary of superordinate and assist and address sub-goals, with variable orientation.

**TABLE 1:** Summary of approach- and avoidance-oriented superordinate and sub-goals.

	Approach-oriented	Avoidance-oriented
Superordinate goal	Approach A	Avoid X
Assist sub-goal	Approach B in order to (superordinate goal)	Avoid Y in order to (superordinate goal)
Address sub-goal	Approach C in order to address the pitfalls of pursuit (of the superordinate goal)	Avoid Z in order to address the pitfalls of pursuit (of the superordinate goal)

## Sub-goals to Support Avoiding COVID-19

This assist-address distinction may be applied to avoiding COVID-19. During the pandemic, many have committed to the superordinate goal of avoiding COVID-19, and have achieved this aim by following CDC recommendations, such as: “Wear a mask”; “Wash your hands often”; “Avoid crowds and poorly ventilated spaces”; “Stay 6 feet away from others”; “Get Vaccinated” (How to Protect Yourself & Others, 2021). Each of these items can be considered sub-goals, specifically assist sub-goals, as each is pursued primarily to meet the aim of the superordinate goal; that is, each sub-goal is pursued in order to avoid COVID-19. These goals also utilize both approach and avoidance to support the avoidance-oriented superordinate goal (avoid COVID-19). Specifically, the described actions in the sub-goals are approach-oriented (wearing masks, washing hands, and getting vaccinated) and avoidance-oriented (avoiding crowds, avoiding certain spaces, and staying 6 feet away).

Avoiding COVID-19 may also involve address sub-goals. These sub-goals, if approach-oriented, can harness the benefits of approach motivation, even while sub-serving an over-arching avoidance aim. For example, address sub-goals may be proposed that foster psychological well-being, thus supporting continued persistence in goal pursuit and goal attainment. These address sub-goals can be approach-oriented to take advantage of the psychological benefits associated with this goal structure (pursuing a positive object). For example, sub-goals such as promoting mental health through online social interaction and promoting physical health through home or safe outdoor exercise are examples of address sub-goals. Though not directly related to the primary objective of avoiding COVID-19, these sub-goals promote goal pursuit and goal attainment of the primary objective by addressing the negative costs of avoidance motivation (the avoidance tax), like reduced well-being. In other words, these sub-goals are pursued primarily to address the limitations of avoidance motivation, and not to achieve the aim of the superordinate goal.

## Motivational Match vs. Mismatch—Different Theoretical Approaches

Our perspective on goal hierarchies may be contrasted with another prominent approach to motivation and self-regulation—regulatory focus theory (RFT). Like our own conceptual approach, RFT highlights the importance of distinguishing between goals focused on approaching gains (promotion-focused) and goals focused on avoiding losses (prevention-focused; Higgins, 1998). Also like our own conceptual approach, RFT posits that goal regulation is hierarchical in nature with, for example, promotion-focused superordinate goals supported by both promotion-focused and prevention-focused sub-goals (Scholer et al., 2019). The key difference is that RFT emphasizes the benefits of a regulatory match between hierarchical levels (e.g., a prevention-focused sub-goal serving a prevention-focused superordinate goal), whereas our approach emphasizes that mismatches between hierarchical levels are commonly optimal when the superordinate goal is avoidance-based (e.g., an approach sub-goal serving an avoidance superordinate goal). Regarding the pandemic, we believe that the superordinate goal of avoiding COVID-19 is often optimally supported by approach rather than avoidance sub-goals, assist and address alike.

## Discussion

Goals exist within a hierarchical structure, with superordinate goals supported by sub-goals. Sub-goals can be classified as assist or address, each supporting superordinate goal attainment by direct (assist) or indirect (address) means. These sub-goals may be approach- or avoidance oriented, and need not match the orientation of the superordinate goal. In fact, approach-oriented sub-goals may prove beneficial to pair with superordinate avoidance goals, as avoidance motivation can deplete energy and undermine well-being. To offset this avoidance tax, approach-oriented sub-goals (approach IN ORDER TO avoid) can be developed and utilized to tap into the psychological benefits of approach motivation. During the current pandemic, many have sought to avoid COVID-19 (superordinate goal) through various means (sub-goals). Future research is needed to test if approach-oriented sub-goals, particularly address sub-goals that promote mental and physical well-being, can offer a silver-lining during a period of illness and anxiety (be it another variant of COVID-19 or a subsequent disease) and help individuals not only avoid sickness, but also thrive.

## Author Contributions

Both authors listed have made a substantial, direct, and intellectual contribution to the work, and approved it for publication.

## Conflict of Interest

The authors declare that the research was conducted in the absence of any commercial or financial relationships that could be construed as a potential conflict of interest.

## Publisher’s Note

All claims expressed in this article are solely those of the authors and do not necessarily represent those of their affiliated organizations, or those of the publisher, the editors and the reviewers. Any product that may be evaluated in this article, or claim that may be made by its manufacturer, is not guaranteed or endorsed by the publisher.
